# EBV-Negative Monomorphic B-Cell Posttransplant Lymphoproliferative Disorder with Marked Morphologic Pleomorphism and Pathogenic Mutations in* ASXL1*,* BCOR*,* CDKN2A*,* NF1*, and* TP53*

**DOI:** 10.1155/2017/5083463

**Published:** 2017-04-10

**Authors:** Agata M. Bogusz

**Affiliations:** Department of Pathology and Laboratory Medicine, Hospital of the University of Pennsylvania, Philadelphia, PA 19104-4283, USA

## Abstract

Posttransplant lymphoproliferative disorders (PTLDs) are a diverse group of lymphoid or plasmacytic proliferations frequently driven by Epstein-Barr virus (EBV). EBV-negative PTLDs appear to represent a distinct entity. This report describes an unusual case of a 33-year-old woman that developed a monomorphic EBV-negative PTLD consistent with diffuse large B-cell lymphoma (DLBCL) 13 years after heart-lung transplant. Histological examination revealed marked pleomorphism of the malignant cells including nodular areas reminiscent of classical Hodgkin lymphoma (cHL) with abundant large, bizarre Hodgkin-like cells. By immunostaining, the malignant cells were immunoreactive for CD45, CD20, CD79a, PAX5, BCL6, MUM1, and p53 and negative for CD15, CD30, latent membrane protein 1 (LMP1), and EBV-encoded RNA (EBER). Flow cytometry demonstrated lambda light chain restricted CD5 and CD10 negative B-cells. Fluorescence* in situ* hybridization studies (FISH) were negative for* cMYC*,* BCL2,* and* BCL6* rearrangements but showed deletion of* TP53* and monosomy of chromosome 17. Next-generation sequencing studies (NGS) revealed numerous genetic alterations including 6 pathogenic mutations in* ASXL1, BCOR, CDKN2A, NF1,* and* TP53*(x2) genes and 30 variants of unknown significance (VOUS) in* ABL1, ASXL1, ATM, BCOR, BCORL1, BRNIP3, CDH2, CDKN2A, DNMT3A, ETV6, EZH2, FBXW7, KIT, NF1, RUNX1, SETPB1, SF1, SMC1A, STAG2, TET2, TP53, *and* U2AF2.*

## 1. Introduction

Posttransplant lymphoproliferative disorders (PTLDs) are lymphoid and plasmacytic proliferations that arise in the setting of immunosuppression in a recipient of a solid organ transplant (SOT) or hematopoietic stem cell transplant (HSCT) [1]. PTLDs affect 1–25% of posttransplant patients, with the highest incidents for intestinal and multiorgan transplant, followed by heart and lung transplants [2]. The revised 2016 World Health Organization (WHO) categorizes PTLDs into the following categories: plasmacytic hyperplasia PTLD, infectious mononucleosis PTLD, florid follicular hyperplasia PTLD, polymorphic PTLD, monomorphic PTLD (B- and T-/NK-cell types), and classical Hodgkin (cHL) lymphoma PTLD [3]. The vast majority of PTLDs are of B-cell origin and are usually associated with Epstein-Barr virus (EBV) infection; however a significant subset are EBV-negative [1, 4, 5]. Early onset PTLDs are typically Epstein-Barr virus- (EBV-) driven lymphoproliferations and may be polyclonal or oligoclonal, whereas late onset ones are typically monoclonal lymphoid malignancies that can lack EBV association. The pathogenesis of non-EBV-related PTLD may be similar to non-Hodgkin's lymphomas (NHL) [6]. EBV-negative PTLD has been proposed to be a distinct entity and typically presents as a late complication of transplantation with a median of 50–60 months [5, 7–10]. EBV-negative PTLDs typically display monomorphic morphology [1]. Here we present a rare case of EBV-negative PTLD occurring more than a decade after solid organ transplant (SOT) and presenting with a large variety of morphologies of the malignant cells and numerous genetic alterations comprising 6 pathogenic mutations (*ASXL1*,* BCOR, CDKN2A, NF1, *and* TP53*x2) and 30 variants of unknown significance (VOUSs).

## 2. Materials and Methods

### 2.1. Histology and Immunohistochemistry

Formalin-fixed paraffin-embedded (FFPE) tissue sections were stained with hematoxylin and eosin (H&E) according to manufacturer's instructions. Immunohistochemical staining was performed on 4 *μ*m tissue sections using an Autostainer (Leica BOND platform, Buffalo Grove, IL) according to manufacturer's instructions. Briefly, sections were deparaffinized in xylene and graded alcohols. Detection of the antibodies was performed using a chromogenic substrate, diaminobenzene (Dako).

### 2.2. Molecular Analysis for Clonality

DNA was extracted from FFPE small bowel tumor tissue and analyzed for clonality as described previously [11]. Briefly, PCR amplification was performed with two sets of fluorescently labeled primers (InVivoScribe Technologies) that hybridize to a conserved V-framework, framework 2 (FR2), and framework 3 (FR3) regions and the conserved J-region of immunoglobulin heavy chain* (IGH)* gene. The PCR products were subsequently size-separated by capillary electrophoresis on a 3500xL Genetic Analyzer (Life Technologies). Data were analyzed (GeneMapper v5.0 software) and examined for peak patterns consistent with a clonal expansion.

### 2.3. Fluorescence In Situ Hybridization (FISH) Analysis

FISH was performed on 3 *μ*m FFPE tissue sections using the MYC break-apart probe, BCL6 break-apart probe, BCL2 break-apart probe, and TP53/NF1 probes (all from Metasystems Group, Inc.) according to the manufacturers' instructions. Briefly, slides were deparaffinized using xylene incubation (×3), followed by ethanol wash steps (100%, 70%). The slides were treated with Dako pretreatment solution (Dako, Inc., K5799) prior to hybridization, followed by digestion with pepsin (37°C, 15 min). Slides were then dehydrated in ethanol (70, 85, and 100%) and dried and the FISH probes were added for incubation overnight. The next day, the slides were washed, counterstained with DAPI, manually visualized, and scored.

### 2.4. Gene Mutation Analysis

Mutational analysis of FFPE tissue samples was performed by the University of Pennsylvania at the Center for Personalized Diagnostics as described previously [11]. The genes sequenced were part of a custom, targeted next-generation sequencing amplicon panel testing for 68 hematologic malignancy-associated genes* (ABL1, ASXL1, ATM, BCOR, BCORL1, BIRC3, BRAF, CALR, CBL, CDKN2A, CEBPA, CSF1R, CSF3R, DDX3X, DNMT3A, ETV6, EZH2, FAM5C, FBXW7, FLT3, GATA2, GNAS, HNRNPK, IDH1, IDH2, IL7R, JAK2, KIT, KLHL6, KRAS, MAP2K1, MAPK1, MIR142, MPL, MYC, MYCN, MYD88, NF1, NOTCH1, NOTCH2, NPM1, NRAS, PDGFRA, PHF6, POT1, PRPF40B, PTEN, PTPN11, RAD21, RIT1, RUNX1, SETBP1, SF1, SF3A1, SF3B1, SMC1A, SRSF2, STAG2, TBL1XR1, TET2, TP53, TPMT, U2AF1, U2AF2, WT1, XPO1, ZMYM3,* and* ZRSR2)* (TruSeq Custom Amplicon, Illumina Inc.) based on previously described analyses [12, 13]. A custom bioinformatics pipeline was utilized to detect alterations [14] and manual data review was performed with variants compared with our knowledgebase and online databases for further curation, using human reference sequence UCSC build hg 19 (NCBI build 37.1) for comparison. Single nucleotide polymorphisms (SNPs) with a minor allele frequency (MAF) > 0.1% were classified as benign and were not reported based on the Exome Variant Server (http://evs.gs.washington.edu/EVS), the ExAC browser (http://exac.broadinstitute.org), and dbSNP. Reported variants used nomenclature that is based on the Human Genome Variation Society nomenclature guidelines (http://www.hgvs.org/mutnomen) and internally categorized into five categories (benign, likely benign, variant of uncertain significance, likely pathogenic, and pathogenic); the categories “likely benign,” “variant of uncertain significance,” and “likely pathogenic” were reported as variants of uncertain significance (VOUSs).

## 3. Case Presentation

A 33-year-old female presented with progressive cramps, emesis, and alternating constipation and diarrhea. The patient received a heart and unilateral lung transplant 13 years prior to presentation for treatment of end-stage congenital heart disease (single ventricle, dextrocardia, and severe pulmonary stenosis). Her posttransplant course was complicated by severe cytomegalovirus (CMV) pneumonia and allograft dysfunction, including an acute rejection of her heart within three months and an episode of lung rejection seven years after transplant. The patient was subsequently stable on immunosuppression (azathioprine, 50 mg oral, 3 times a day, and prednisone, 5 mg oral, once a day). The patient was referred for evaluation at our institution. A computed tomography (CT) scan of the abdomen showed thickening of the jejunum. Surgical resection revealed a 9 cm exophytic tumor in the small bowel.

## 4. Pathologic Findings

**Figure 1:**
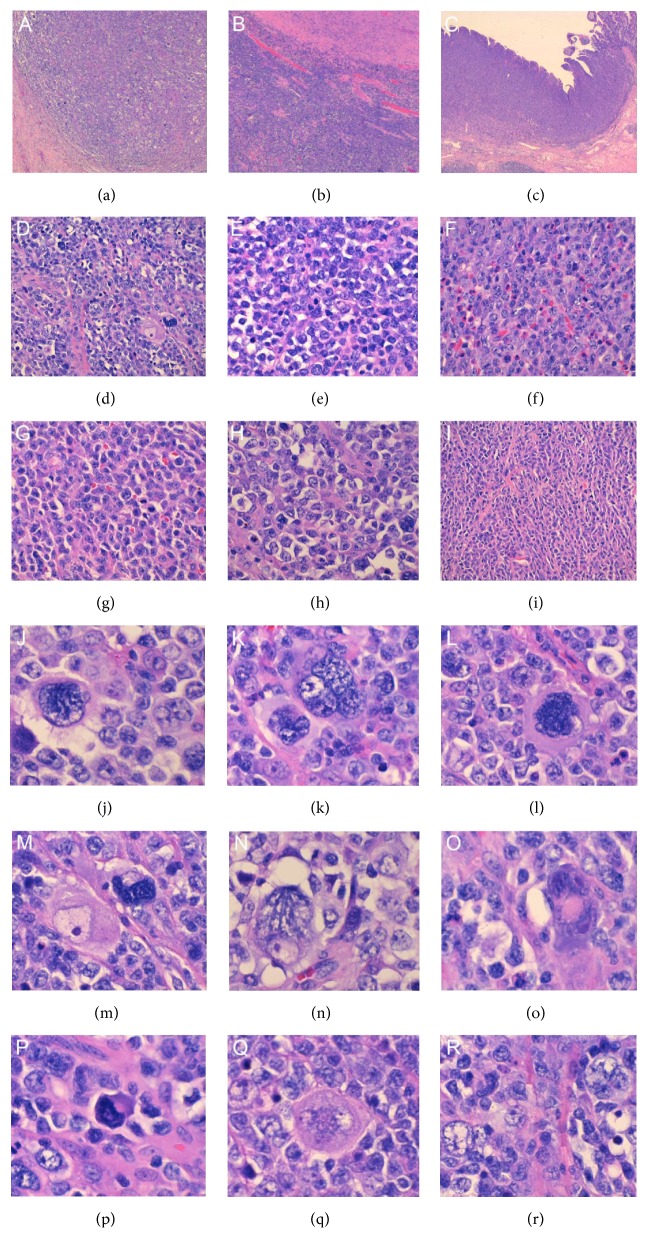
Histological findings of the small bowel tumor. Low power examination (50x) shows (a) nodular cellular areas with scattered large atypical cells surrounded by thick bands of fibrosis as well as (b) monomorphic areas with dense blue cells and more clear areas and foci of necrosis and (c) monomorphic areas with increased eosinophils in the lamina propria. Higher power examination (200x) of the different areas of the specimen reveal a variety of morphologies with (d) pleomorphic areas with many bizarre large atypical cells, (e) areas with monotonous medium- to large-sized cells, (f) areas with increased infiltrating eosinophils, (g) cells with plasmacytoid appearance, (h) areas with clear large cells, and (i) areas with spindle-shaped cells with somewhat plasmacytoid features. High power examination (400x) of areas seen in (i) and (d) shows the variety of morphologies of the large atypical cells (j-r) with (j) lacunar cells, (k) multinucleated cells, (l) markedly hyperchromatic cells with dense eosinophilic cytoplasm, (m,n) bizarre cells with eosinophilic nucleoli, (o) Reed-Sternberg-like cells with smudgy eosinophilic nucleoli and dense ampophilic cytoplasm, (p) mummified cells, (q) large atypical cells with multiple clear nuclei, and (r) popcorn-like cells with small nucleoli.

Histological examination by hematoxylin and eosin (H&E) staining (Figure 1) revealed a large morphologic heterogeneity in this specimen. Under low power there were present nodular cellular areas intercepted by thick bands of fibrosis (Figure 1(a)) reminiscent of nodular sclerosis type cHL, areas with more monomorphic large immunoblast-like cells and foci of necrosis (Figure 1(b)) and areas with monomorphic appearance with admixed abundant eosinophils (Figure 1(c)). Higher power examination of the nodular areas revealed numerous large, highly atypical cells (Figure 1(d)) with a variety of morphologies (Figures 1(j)–1(r)) including lacunar cells, multinucleated cells, markedly hyperchromatic cells, mummified cells, Reed-Sternberg-like cells, and popcorn cells. Higher power examination of the monomorphic appearing areas revealed a remarkable diversity of morphology, including areas with monotonous medium to large cells (Figure 1(e)), increased infiltrating eosinophils (Figure 1(f)), prominence of plasmacytoid cells (Figure 1(g)), clear large cells (Figure 1(h)), and spindle-shaped cells (Figure 1(i)).

**Figure 2:**
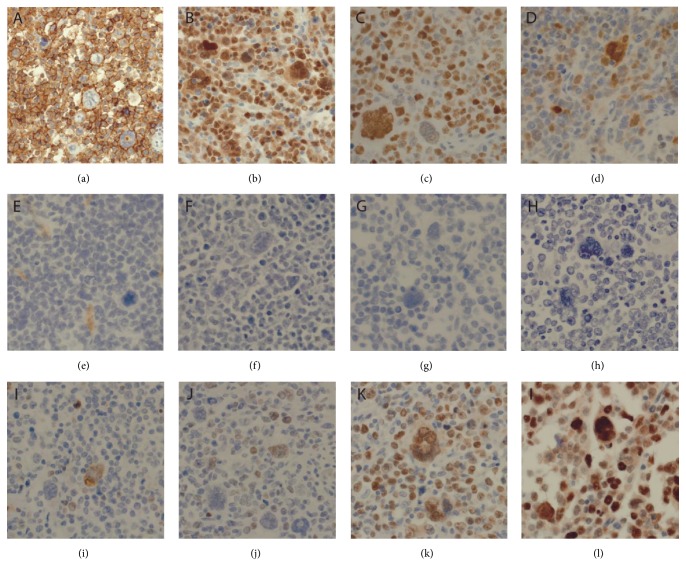
Immunophenotypic findings of the small bowel tumor. The tumor cells are immunoreactive for (a) CD20, (b) PAX5, (c) MUM1, and (d) BCL6 (major subset). The tumor cells are negative for (e) CD10, (f) CD30, (g) CD15, and (h) EBER. (i) Staining for BCL2 and (j) cMYC shows only occasional positive cells. (k) Ki67 staining reveals that high proliferation index is approximately 70%. (l) The staining for p53 was strongly positive.

Immunohistochemistry revealed that the malignant cells, including the very large atypical cells, were immunoreactive for CD45, CD20 (Figure 2(a)), CD79a, PAX5 (Figure 2(b)), BCL6 (Figure 2(c)), and MUM1 (predominantly in the larger cells, Figure 2(d)) and were negative for CD3, CD5, CD10 (Figure 2(e)), CD30 (Figure 2(f)), CD15 (Figure 2(g)), LMP1, EBER (Figure 2(h)), and CMV. BCL2 was positive only in rare cells (Figure 2(i)) and cMYC (Figure 2(j)) was only focally positive but was overall negative (<40% positive cells, Figure 2(j)). The proliferation index as determined by Ki67 staining was high at 70% (Figure 2(k)). Staining for p53 was strongly and diffusely positive (Figure 2(l)).

Flow cytometry demonstrated a population of variably sized surface lambda light chain restricted CD5 and CD10 negative B-cells that represented the predominant population of monotonous large immunoblast-like B-cells. A diagnosis was rendered of monomorphic PTLD (B-cell type) with features of a diffuse large B-cell lymphoma, EBV-negative with pleomorphic, HL/RS-like cells.

**Figure 3:**
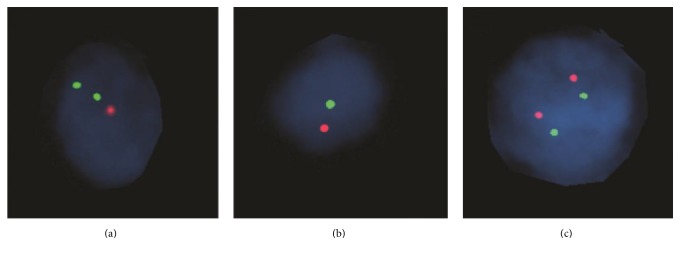
FISH analysis for* TP53* deletion. FISH studies performed on formalin-fixed paraffin-embedded sections of the small bowel tumor using TP53/NF1 probe revealed (a) deletion of* TP53* in 14/100 cells and (b) monosomy of chromosome 17 in 20/100 cells. (c) Normal cell.

Fluorescence* in situ* hybridization studies (FISH) were negative for* cMYC*,* BCL2*, and* BCL6* rearrangements but revealed deletion of* TP53* in 14/100 cells (Figure 3(a)) and monosomy of chromosome 17 in 20/100 cells (although these results fall within the range of the cutoff of 20–30% on paraffin tissue) (Figure 3(b)) as compared to normal cells (Figure 3(c)).

**Figure 4:**
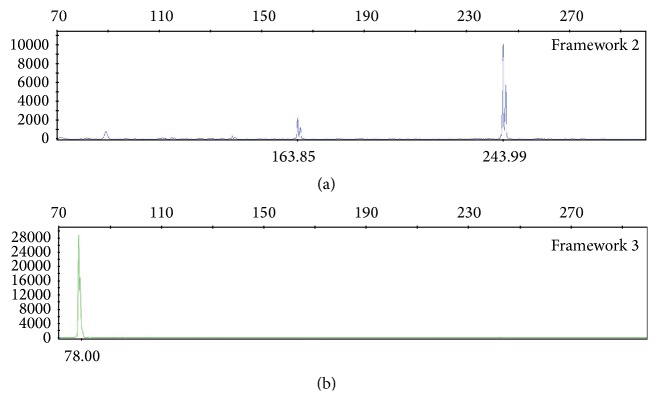
*IGH* PCR analysis of the small bowel tumor.* IGH* PCR analysis using primers for framework 2 (FR2) region identified two clonal peaks at approximately 163.85 bp and 243.99 bp (a). Primers targeting framework 3 (FR3) region identified a 78 bp peak (b).

Molecular studies for* IGH* gene rearrangement performed on the DNA extracted from the small bowel tumor revealed a 163.85-base pair (bp) peak and a 243.99 bp peak in framework 2 (FR2) as well as a 78 bp peak in framework 3 (FR3) (Figure 4) confirming a clonal process.

Next-generation sequencing (NGS) studies revealed numerous genetic alterations including 6 pathogenic mutations in* ASXL1, BCOR, CDKN2A, NF1,* and* TP53*(x2) genes and 30 variants of unknown significance (VOUSs) in* ABL1, ASXL1, ATM, BCOR, BCORL1, BRNIP3, CDH2, CDKN2A, DNMT3A, ETV6, EZH2, FBXW7, KIT, NF1, RUNX1, SETPB1, SF1, SMC1A, STAG2, TET2, TP53,* and* U2AF2* (Table 1).

## 5. Clinical Follow-Up

Staging of the patient confirmed that her disease was confined to the small bowel. The patient's immunosuppression was decreased and following recovery from surgery she received rituximab and R-CY/VP16 (rituximab-cyclophosphamide/etoposide) with no response and subsequently responded to R-CHOP (4 cycles). The patient appeared in complete remission by CT scan ten months after surgery but developed pleural effusion following the 4th cycle of R-CHOP and was also found to have retraction of the transplanted lung and anemia (Hg 9.5 g/dL). While receiving the next cycle of R-CHOP and transfusion 4 weeks later, the patient went into cardiac arrest and expired at the hospital. An autopsy was not requested. The cause of death was listed as shock, respiratory failure, hypotension, and asystole related to acute transplant rejection.

## 6. Discussion

PTLDs are a clinically and morphologically heterogeneous group of diseases that occur after organ transplant. Histologically, PTLDs comprise a spectrum of lymphoid proliferations that range from polyclonal expansions to overt lymphomas [3]. This case is characterized by very late onset and EBV negativity. EBV-negative PTLDs typically present years after transplantation and display monomorphic morphology [15]. In rare cases a progression from an EBV-positive to EBV-negative neoplasm has been suspected [16]. The prognosis of EBV-negative PTLD appears to be similar to EBV-positive cases [17]. The reduction of immunosuppressive therapy is considered the first step in treating PTLD; however patients with poor prognostic factors, such as late onset disease and EBV negativity, typically require chemotherapy and immunotherapy [15]. It has been postulated that EBV-negative PTLD represents a distinct entity [5, 8]. In support of this hypothesis, gene expression profiling studies have revealed clear differences between EBV-positive and EBV-negative PTLDs [10, 18].

This case is unique in that several distinct morphologies coexist within this monomorphic PTLD including a nodular sclerosis Hodgkin lymphoma- (HL-) like component and large B-cell lymphoma component with diverse morphology (Figure 1). The large atypical HL/RS-like cells within the HL-like component were negative for CD15 and CD30 and immunoreactive for CD45 and CD20 (Figure 2) ruling out cHL PTLD and supporting the diagnosis of PTLD, DLBCL type. Since the patient's tumor was negative for CD10 and positive for BCL6 and MUM1, it can be classified as the more aggressive non-GCB (germinal center B-cell) type DLBCL [19].

NGS studies revealed a large number of genetic alterations in the patient's tumor (Table 1) with most of the altered genes involved in chromatin remodeling and DNA repair. This included 2 pathogenic mutations and 1 VOUS in* TP53*. A recent paper demonstrates that EBV-negative PTLDs frequently contain* TP53* mutations implicating p53 role in the disease process [9]. The pathogenesis of EBV-negative PTLD is not well understood but frequent* TP53* mutations might be one of the contributory factors [9]. Staining for p53 protein was uniformly strong in the tumor, correlating well with the presence of mutations. Expression of p53 in de novo DLBCL was shown to be correlated with inferior outcome [20, 21]. In addition to* TP53* mutations, FISH studies of the patient's tumor showed deletion of* TP53* in 14/100 cells and monosomy of chromosome 17 in 20/100 cells (Figure 3).

Other pathogenic mutations in this tumor affected* ASXL1, BCOR, CDKN2A,* and* NF1* genes (Table 1).* ASXL1* is one of the most mutated genes in myeloid neoplasms including chronic myelomonocytic leukemia (CMML), acute myeloid leukemia (AML), myelodysplastic syndrome (MDS), and myelodysplastic/myeloproliferative neoplasm (MDS/MPN) [22] and only rare mutations have been reported in lymphoid malignancies such as chronic lymphocytic leukemia (CLL) [23]. Mutations in* ASXL1* are generally associated with poor prognosis in myeloid malignancies [24].* BCOR* encodes BCL6 interacting corepressor. BCL6 is a zinc-finger transcriptional repressor and key regulator of germinal center reaction that is frequently translocated and hypermutated in DLBCL [25]. However, NGS studies of 388 cases of B-cell lymphomas revealed only one Burkitt lymphoma case with a missense* BCOR* mutation (S1295T) [26]. Interestingly, frequent* BCOR* aberrations were reported in extranodal NK/T-cell lymphoma, nasal type [26]. In addition to one pathogenic mutation in* BCOR,* we have detected three VOUSs in* BCOR* and two VOUSs in* BCORL1* gene.* BCOR* and* BCORL1* are homologous X-linked genes that act as corepressors that were found to be recurrently mutated in AML. About 50% of* BCOR/BCORL1*-mutated cases also carry* DNTM3A* mutations and we have detected a VOUS in* DNTM3A* gene (Table 1).* CDKN2A* mutations have been previously reported in DLBCL [27]. Interestingly, proteins encoded by* TP53* and* CDKN2A* are components of the p53 pathway and it has been previously reported that alterations in these genes are independent in DLBCL, providing additional tumor growth advantage [28].* NF1* mutations have been reported in rare cases of orbital DLBCL [29].

The VOUSs detected in this patient's sample (Table 1) included many genes that are typically mutated in myeloid malignancies* (ABL1, ASXL1, BCOR, BCORL1, BRNIP3, DNMT3A, ETV6, EZH2, KIT, RUNX1, SETPB1, SF1, SMC1A, STAG2, TET2*, and* U2AF2)* and not commonly seen in lymphoid malignancies. The significance of these findings is unclear but may suggest the pathogenesis of EBV-negative monomorphic PTLD is much more complex than that of EBV-driven PTLD. It has been proposed to stratify PTLD according to the histological subtype and EBV status in future clinical trials so as to better understand the mechanisms underlying the PTLD lymphomagenesis [30].

## Figures and Tables

**Table 1:** Genetic alterations comprising pathogenic mutations and VOUSs (variants of unknown significance) detected in the patient's PTLD specimen.

Gene	Protein change	cDNA change	Categorization	Allele frequency
*ABL1*	p.G882Afs^*∗*^12	c.2648delG	VOUS	36.3
***ASXL1***	p.G645Vfs^*∗*^58	**c.1934delG**	**Pathogenic**	**18.63**
*ASXL1*	p.A1016V	c.C3047T	VOUS	32.09
*ASXL1*	p.C1240F	c.G3719T	VOUS	42.79
*ATM*	p.E871K	c.G2611A	VOUS	37.24
***BCOR***	**p.P1621Qfs** ^*∗*^ **53**	**c.4862delC**	**Pathogenic**	**31.07**
*BCOR*	p.L1333M	c.C3997A	VOUS	57.17
*BCOR*	p.P407L	c.C1220T	VOUS	52.6
*BCOR*	p.P178L	c.C533T	VOUS	19.94
*BCORL1*	p.T39M	c.C116T	VOUS	23.36
*BCORL1*	p.R500H	c.G1499A	VOUS	63.21
*BRINP3*	p.A437T	c.G1309A	VOUS	36.36
*CDH2*	p.L870M	c.T2608A	VOUS	34.9
***CDKN2A***	**p.R58** ^*∗*^	**c.C172T**	**Pathogenic**	**38.3**
*DNMT3A*	p.A70V	c.C209T	VOUS	35.05
*ETV6*	p.R259W	c.C775T	VOUS	38.87
*EZH2*	p.Y330C	c.A989G	VOUS	53.56
*FBXW7*	p.R441Q	c.G1322A	VOUS	45.79
*KIT*	p.P468L	c.C1403T	VOUS	36.26
*KIT*	p.N564D	c.A1690G	VOUS	35.63
*NF1*	p.P464L	c.C1391T	VOUS	38.12
***NF1***	**p.Q2147** ^*∗*^	**c.6439_6441delinsTAG**	**Pathogenic**	**35.66**
*RUNX1*	p.R250C	c.C748T	VOUS	38.67
*SETBP1*	p.R54H	c.G161A	Likely benign	45.23
*SETBP1*	p.G1392S	c.G4174A	VOUS	39.64
*SETBP1*	p.R589^*∗*^	c.C1765T	VOUS	38.75
*SF1*	p.N200Y	c.A598T	VOUS	39.27
*SMC1A*	p.R1066H	c.G3197A	VOUS	25.34
*STAG2*	p.S853R	c.T2559A	VOUS	14.12
*TET2*	p.A347V	c.C1040T	VOUS	38.03
*TET2*	p.V1900I	c.G5698A	VOUS	36.76
***TP53***	**p.G245S**	**c.G733A**	**Pathogenic**	**38.4**
*TP53*	p.E56K	c.G166A	VOUS	40.77
***TP53***	**p.R306** ^*∗*^	**c.C916T**	**Pathogenic**	**40.02**
*U2AF2*	p.R44H	c.G131A	VOUS	20.15

*ABL1*: Abelson Tyrosine Kinase; *ASXL1*: Additional Sex Combs-Like 1; *BCOR*: BCL6 Corepressor; *BCORL1*: BCL6 Corepressor-Like 1; *BRINP3*: BMP/Retinoic Acid Inducible Neural Specific 3; *CDH2*: Cadherin 2; CDKN2A: cyclin-dependent kinase inhibitor 2A; *DNMT3A*: DNA methyltransferase 3 alpha; *ETV6*: ETS (erythroblast transformation-specific) variant 6; *EZH2*: enhancer of zeste 2 polycomb repressive complex 2 subunit; *FBXW7*: F-Box and WD Repeat Domain Containing 7; *KIT*: KIT Protooncogene Receptor Tyrosine Kinase; *NF1*: Neurofibromin 1; *RUNX1*: Runt Related Transcription Factor 1; *SETBP1*: SET Binding Protein 1; *SF1*: Splicing Factor 1: *SMC1A*: Structural Maintenance of Chromosomes 1A; *STAG2*: Stromal Antigen 2: *TET2*: Tet Methylcytosine Dioxygenase 2: *TP53*: Tumor Protein P53; *U2AF2*: U2 Small Nuclear RNA Auxiliary Factor 2.
